# BarWare: efficient software tools for barcoded single-cell genomics

**DOI:** 10.1186/s12859-022-04620-2

**Published:** 2022-03-27

**Authors:** Elliott Swanson, Julian Reading, Lucas T. Graybuck, Peter J. Skene

**Affiliations:** 1 Allen Institute for Immunology, Seattle, WA USA; 2 Present Address: Department of Genome Sciences, University of Washington School of Medicine, Seattle, WA USA

**Keywords:** Single-cell RNA-seq, Cell hashing, Demultiplexing, Genomics

## Abstract

**Background:**

Barcode-based multiplexing methods can be used to increase throughput and reduce batch effects in large single-cell genomics studies. Despite advantages in flexibility of sample collection and scale, there are additional complications in the data deconvolution steps required to assign each cell to their originating samples.

**Results:**

To meet computational needs for efficient sample deconvolution, we developed the tools BarCounter and BarMixer that compute barcode counts and deconvolute mixed single-cell data into sample-specific files, respectively. Together, these tools are implemented as the BarWare pipeline to support demultiplexing from large sequencing projects with many wells of hashed 10x Genomics scRNA-seq data.

**Conclusions:**

BarWare is a modular set of tools linked by shell scripting: BarCounter, a computationally efficient barcode sequence quantification tool implemented in C; and BarMixer, an R package for identification of barcoded populations, merging barcoded data from multiple wells, and quality-control reporting related to scRNA-seq data. These tools and a self-contained implementation of the pipeline are freely available for non-commercial use at https://github.com/AllenInstitute/BarWare-pipeline.

**Supplementary Information:**

The online version contains supplementary material available at 10.1186/s12859-022-04620-2.

## Background

**Fig. 1:**
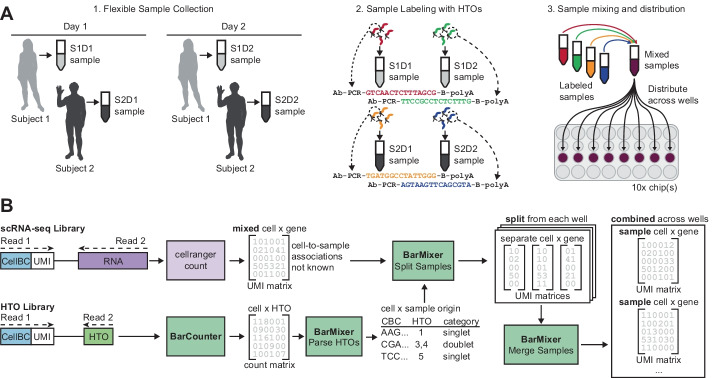
Overview of the Cell Hashing workflow. **A** Sample collection and distribution overview. Cell Hashing allows flexible sample collection, including collection of multiple samples over time from the same donor. Each sample can then be thawed and stained with antibodies conjugated to Hash Tag Oligos (HTOs), each of which contains a unique barcode sequence. Once stained, samples can be mixed and distributed across wells for processing to reduce batch effects. **B** BarWare pipeline overview. After sequencing, two libraries are generated for each 10x Genomics Chromium well: a RNA library, and a HTO library. RNA libraries are aligned and converted UMI count matrices containing cell x gene counts (top), and HTO libraries are counted by Barcounter to generate cell x HTO counts (bottom). BarMixer is then used to convert HTO counts to sample assignments for each cell. BarMixer then combines sample assignments with the scRNA-seq to split sample data within each well, and finally merges data from each sample from all wells to generate sample-specific output. CellBC, Cell Barcode; UMI, Unique Molecular Identifier; HTO, Hash Tag Oligonucleotide

The use of single-cell genomics has rapidly expanded due to high throughput, widely used commercial technologies. Microfluidic droplet based platforms [11, 21] are commonly used for single-cell RNA sequencing (scRNA-seq) due to their ease of use and ability to sequence tens to hundreds of thousands of cells per experiment, and competitive cost per cell. However, droplet-based methods also have inherent challenges including multiple cell capture (multiplets) and well-to-well variation. A strategy to address these issues is Cell Hashing [17] Fig. 1), in which antibodies against near-ubiquitously expressed surface proteins are conjugated to barcoded Hash Tag Oligos (HTO) used to uniquely label samples (Fig. 1A). This allows samples to be mixed and processed simultaneously in the same well to enable increased cell loading and explicit doublet detection, even when multiple samples originate from the same subject. In addition, samples can be mixed and loaded across multiple wells and/or microfluidic chips, eliminating common sources of technical variation and mitigating the risk of sample dropout due to loss of any single well (Fig. 1A). We have recently implemented this approach at scale for multimodal immunomonitoring of patient samples to study the immune system [5].

With the advantages of Cell Hashing come additional complications related to data processing: samples are no longer directly associated with a single set of well indices and must be demultiplexed at two levels, both by well and by sample, for downstream analysis (Fig. 1B). Previously published tools for barcode quantification were very flexible but slow and did not include the ability to easily repartition count matrices for each sample. To implement Cell Hashing at scale, we developed BarWare, an efficient and comprehensive pipeline consisting of two modular tools linked by shell scripting: BarCounter, for fast, efficient tabulation of HTO counts per cell barcode, and BarMixer, an R package that provides code to quickly redistribute samples across wells and report results and quality control (QC) metrics in user-friendly reproducible RMarkdown reports.

We show that BarCounter outperforms other Hash Tag Oligo (HTO) counting tools and demonstrate the BarWare Cell Hashing pipeline using a large benchmark dataset generated by progressive overloading of the 10x Chromium v3 3′ RNA-seq assay. These capabilities, combined with an emphasis on automated quality control reporting, make BarWare a scalable, user-friendly, and comprehensive toolkit for Cell Hashing that can be efficiently applied to large-scale sequencing projects with many wells of 10x 3′ RNA-seq data.

## Implementation

### Efficient barcode counting with BarCounter

**Fig. 2:**
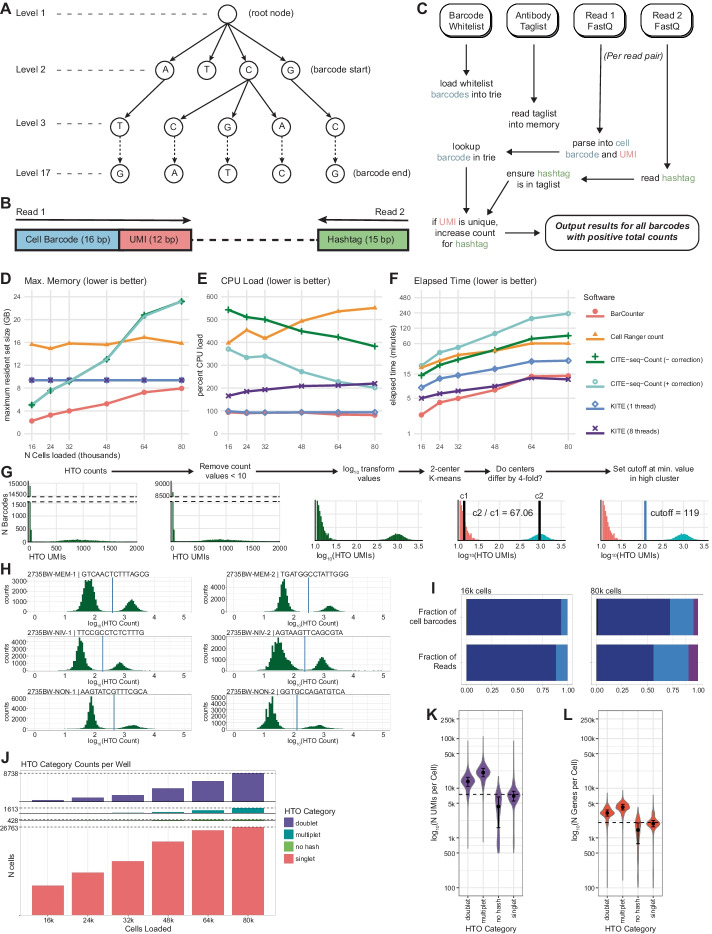
Implementation of BarCounter and BarMixer. **A** Trie data structures are used in BarCounter to efficiently tabulate barcode frequencies. **B** Diagram of HTO read structure. Read 1 contains the 16 bp cell barcode and the 12 bp UMI. Read 2 contains the 15 bp hashtag. **C** Overview of the BarCounter workflow. At runtime, the user provides a barcode whitelist which is loaded into a trie for rapid lookups, a taglist containing all valid hashtag sequences, and paired Read 1 and Read 2 FASTQ files. For each read, checks are performed to verify the cell barcode exists in the barcode trie, and the hashtag sequence is in the taglist. The UMI sequence is checked against a trie and if it is not present, the trie is updated and the counts for the barcode and hashtag combination are incremented. **D–F** Benchmarking comparisons of BarCounter and other available HTO counting algorithms as a function of increasing cell loading per 10x Genomics well: cellranger count (10x Genomics); CITE-seq-Count (with or without barcode correction, [15], KITE, [4] (single-threaded or with 8 threads). **D** Maximum memory usage, **E** Average CPU load, **F** Elapsed time. **G** Overview of the barcode cutoff determination method used by BarMixer: Raw counts generated by BarCounter are clipped to remove low values, then log transformed, and used as input to 2-cluster K-means. If cluster medians are separable, the cutoff is set to the lowest value in the positive cluster. Note broken y-axis in the first two panels. **H–L** Visualizations provided by BarMixer QC reporting notebooks. **H** HTO count histograms (green bars) with cutoff values (blue lines), **I** Fractions of barcodes and reads attributed to singlets (dark blue), doublets (light blue), and multiplets (purple), **J** Counts of cells in each hashing category per well in a batch, **K** Number of UMIs per cell in each HTO category, **L** Number of genes per cell in each HTO category

We identified HTO counting as a significant bottleneck in the processing of Cell Hashing data. In particular we found that a popular and widely used tool, CITE-seq Count [15], scaled poorly to highly overloaded wells both in terms of processing time and memory. As the cost of single-cell sequencing continues to decline, large Cell Hashing and CITE-seq experiments on the order of hundreds of thousands to millions of cells are being generated. To facilitate rapid and parallel processing of large datasets we developed BarCounter: a fast, scalable HTO counting program implemented in C and optimized with speed and memory use in mind. Briefly, BarCounter parses paired-end FASTQ data into cell barcode, Unique Molecular Identifier (UMI), and hashtag sequences, then matches barcodes and hashtags against a user-provided cell barcode whitelist and hashtag sequence list, respectively. To account for sequencing errors, BarCounter allows a single base mismatch in hashtag sequences and a single low quality basecall (Q < 20) mismatch in cell barcodes. BarCounter processes each read independently and utilizes the trie data structure (also known as a prefix tree) to perform cell barcode and UMI lookups in constant time (Fig. 2A–C).

### Assignment of counts to hashed populations

The BarMixer package includes tools to convert raw HTO counts from BarCounter into assignments of each cell to their sample of origin. BarMixer assigns barcodes as “singlet”, “doublet”, “multiplet”, or “no hash” based on dynamically determined UMI cutoffs specific to each hash sequence in each well. For each hashtag, a distribution of HTO counts across all cell barcodes is generated, and a cutoff value delineating positive and negative barcodes is assigned (Fig. 2G and “Methods”). Barcode categories are determined based on the number of positive hashes, e.g. cell barcodes positive for a single hash are classified as singlets. Barcodes are labelled with sample names corresponding to each positive hash. Processing metrics are organized into JavaScript Object Notation (JSON) and HTML reports for convenient automated and visual quality control.

### Distribution of cells with BarMixer

Sample-specific datasets are prepared via BarMixer by performing three key steps. For each well, BarMixer annotates Cell Ranger filtered HDF5 files with QC characteristics and cell metadata. Then, BarMixer uses the sample assignments for each cell to split data into separate HDF5 files by sample. Finally, BarMixer merges data across all processed wells based on the sample assignments. This yields a separate, merged HDF5 file for each sample, a merged HDF5 file for all multiplets, and metric reports in JSON and HTML format. Reports include relevant sequencing QC metrics, alignment distributions by barcode category, UMI and gene count distributions by hashtag, and median count data by both sample and well.

### Progressive cell overloading to assess demultiplexing

**Fig. 3:**
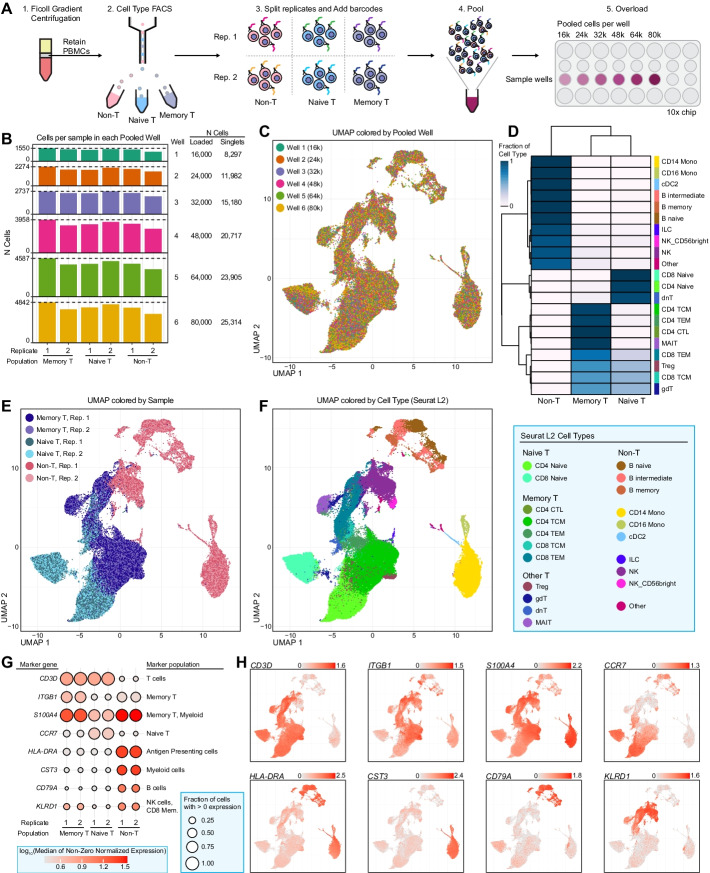
Cell type sorting and progressive overloading to assess overloading and deconvolution. **A** Overview of the workflow for generating the progressively overloaded dataset. PBMCs purified from a Ficoll gradient were sorted into three populations using FACS. Each population was split and stained with a hashing antibody. All samples were then pooled, and were loaded at increasing cell count into the wells of a 10x 3′ scRNA-seq chip. **B** Bar plot showing the cell counts from each population (x-axis) in each well (y-axis). **C** UMAP plot of all singlets (n = 105,395 cells) colored based on which well the mixed samples were loaded into. **D** Heatmap colored based on the fraction of cells from each hashed population assigned to each cell type using Seurat v4.0 label transfer methods. **E** UMAP plot, as in **C**, colored based on the sorted and hashed replicates. **F** UMAP plot, as in **C**, colored based on cell type assignments from Seurat v4.0 label transfer (legend to the right). **G** Dot plot showing the expression of well-known, population-specific marker genes for each replicate. Size corresponds to the fraction of cells in each group with > 0 expression. Color corresponds to the log-transformed median of all non-zero, normalized values in each group. **H** Overlay of the marker gene expression values per cell on the UMAP plot used in panel **C**. Color corresponds to normalized expression values. Color scales are independent per panel, with ranges indicated by the legend at the top-right of each plot

We evaluated the BarWare pipeline and related tools by conducting a progressive cell overloading experiment (Fig. 3). We used fluorescence activated cell sorting (FACS) to separate a sample of peripheral blood mononuclear cells (PBMCs) into naïve T cells, memory T cells, and non-T cell PBMC populations. Each sorted population was divided into two technical replicates for a total of six samples that were stained with commercially available HTO antibodies (BioLegend TotalSeq-A). The six samples were pooled together and loaded into six wells of a 10x Chromium v3 3′ RNA-seq chip at inputs of 16k, 24k, 32k, 48k, 64k, and 80k cells per well (Fig. 3A). Sequencing depth was scaled linearly with predicted cell recovery by well. Hashtag read counts ranged from approximately 40M for 16,000 cell loading to ~ 163M for 80,000 cell loading (Additional file 3: Table S3). This dataset provides a unique test case for HTO counting that is applicable across a wide range of cell numbers and read counts.

## Results and discussion

### Comparison of BarCounter to HTO counting tools

We compared BarCounter to other popular software tools for HTO counting, including CITE-seq-Count (with and without optional UMI correction), Cell Ranger count (10x Genomics), and kallisto indexing and tag extraction (KITE) in both single and multithreaded modes [4]. Some of these methods perform computationally costly Unique Molecular Identifier (UMI) correction because sequencing errors may artificially inflate UMI counts and distort the data. This correction is important for rare transcripts or markers, but commercially available HTO barcode sequences have a universal minimum hamming distance of three bases to ameliorate the risk of hashtag misidentification.

To evaluate the accuracy of BarCounter compared to a method including UMI correction, we ran BarMixer (described below) with HTO counts from either BarCounter or CITE-seq count with UMI correction and compared overlap in barcode classification and sample identification. For each of the six mixed wells, over 99.8% of barcodes identified as singlets were identical between the two methods (Additional file 1: Table S1). Across all wells, 113,414 barcodes were identified as singlets by BarCounter, only 60 of which were identified as doublets by CITE-seq Count. Counts for the top two hashtags for these barcodes differed between the methods by an average of 3.8% and 2.5% respectively, with the majority of barcodes having a count ratio between the top two hashtags greater than three, supporting their classification as singlets (Additional file 2: Table S2). All 113,268 barcodes identified as singlets by both tools had matching sample identity classifications. Therefore, the high dynamic range between positive (bound) and negative (unbound) HTO populations for each cell barcode enables hashtag analysis to be performed without computationally expensive UMI correction with little loss of accuracy in sample identification and doublet detection.

For each Cell Hashing well, we processed HTO FASTQ data using each tool and tracked performance using the Linux “time -v” command. For each well, BarCounter had the lowest memory usage (defined as maximum resident set size), lowest CPU usage, and lowest user (CPU) time. BarCounter was fastest in real (wall clock) time across all comparisons with the exception of the 64,000 and 80,000 cell wells, in which eight-threaded KITE processing was 7% and 15% faster, respectively (Fig. 2D–F). Due to the low-dimensional nature and inherent background signal of HTO data, we opted to output results in the universally readable comma separated values (CSV) format rather than a sparse matrix format. Despite this change, BarCounter outputs were the smallest in terms of disk space across all comparisons (Additional file 4: Table S4).

Based on these performance metrics, we estimate that data from an eight well experiment loading 16,000 cells and sequencing to a depth of 40M reads (~ 2500 reads per cell in this experiment) per well could be processed in parallel on a modest 8 CPU, 20 GB RAM computer in less than five minutes. These results demonstrate that BarCounter is ideally suited for the parallel processing of large Cell Hashing datasets, including when well number, cell recovery, and sequencing depth are high.

### Separation of sample data using BarMixer

We developed a second tool to apply Cell Hashing to samples distributed across multiple wells (Fig. 1B). BarMixer is an R package and set of Rmarkdown notebooks that enables separation of samples within each well (splitting by hash) and reassembly of each sample across all wells into sample-specific output files (merging by hash). First, HTO counts generated by BarCounter are processed to identify a threshold value for each HTO barcode, assign each cell barcode to its corresponding sample(s) as a singlet, doublet, or multiplet (Fig. 2G, “Methods”), and generate an HTML-based report for HTO category counts and cutoffs (including Fig. 2H). Then, HDF5-formatted count matrix results from Cell Ranger count (10x Genomics) are preprocessed to add multiple points of cell metadata, assign each cell barcode with a universally unique identifier (UUIDs) to avoid cell barcode conflicts between wells, and generate a QC report for each well. Next, the HTO category and count data, as well as the metadata-tagged HDF5 count matrix file are used to split each well, create separate HDF5 files for each sample and a separate file for multiplets, and generate a report of sample metadata for each well (including RNA-seq read usage as displayed in Fig. 2I). Finally, the results from each sample across all wells are merged into a single output file per sample, and a final report summarizing HTO categories and RNA-seq QC characteristics is generated for all wells (including Fig. 2J–L). This modular series of steps and reporting allows for rapid assessment of results using the final summary report, as well as step-by-step troubleshooting of each major process in the sample demultiplexing pipeline.

### Evaluation of sample assignments

To evaluate the fidelity of BarWare’s sample assignments, we utilized the Barware pipeline to process a progressive cell overloading dataset, then performed analysis using Seurat v4 [8] to confirm that the samples identified via Cell Hashing and the original FACS sorted populations were in agreement (Fig. 3).

Following simple QC filtering, we performed dimensionality reduction, clustering, and visualized the results using uniform manifold approximation and projection (UMAP). We observed that cells from all eight wells were mixed evenly, there was complete overlap between technical replicates, and that each sorted FACS population showed a high degree of separation from the others (Fig. 3C, E). We then mapped our data onto the reference PBMC CITE-seq dataset described in [8], and transferred the reference cell type labels to our dataset (Fig. 3D, F). As expected, cells with hashes from the non-T cell FACS population were assigned to non-T cell identities (32,069 of 32,199 cells; 99.6%), memory T cell sorts were assigned memory T cell type identities (35,101 of 36,932 cells; 95%), and naïve T cell hashes were most frequently assigned to naïve T cell and double-negative T cell (dnT) identities (30,706 of 36,264 cells; 84.7%) (Fig. 3D–F).

We also visualized cell type-specific marker genes on our UMAP and found highly specific gene expression patterns that support the labelled cell type identities. Expression of CD3D was restricted to the naïve and memory T cell populations, CD14 and MS4A1 (CD20) expression identified classical monocytes and B cells, respectively in the non T cell population, and GNLY was specific to labelled NK and CD8 T cells (Fig. 3E). We divided the T cell compartment into naïve and memory based on expression of CCR7 or S100A4 [7], respectively (Fig. 3F), and found their expression to be mutually exclusive and constrained to the expected FACS populations.

Taken together, the gene expression results show agreement between sample assignments from FACS and Cell Hashing, and confirm that BarWare demultiplexes mixed samples to a high degree of accuracy.

## Conclusion

We have demonstrated the advantages and efficiency of BarWare through its application to a large, multi-well Cell Hashing experiment representing a broad range of cell overloading. BarCounter outperformed other HTO counting tools in terms of speed and computing resources with no decrease in accuracy. BarMixer performs barcode demultiplexing and provides thorough reports detailing QC metrics, and produces merged, sample-specific analysis ready data files along with reports describing the results by sample, by well, and by batch.

In addition, our cell overloading dataset demonstrated the utility of BarWare outputs in simplifying downstream analysis of complex experiments. Merged outputs reduce the number of output files and eliminate manual separation of samples, while maintaining experimental metadata such as the original 10x well, identified hashtag, and barcode classification. BarMixer’s split and merge approach allows analysis of separate samples, independent of the cell pooling performed at the bench, which we have utilized to enable scalable multimodal immunosurveilance studies [5]. We expect this feature to become increasingly beneficial as other research institutes and large consortia scale single-cell data generation to the order of tens of millions of cells. Finally, the cell overloading dataset provided with these tools should be useful in the development of new methods for rapid sample demultiplexing.

BarWare provides a comprehensive set of tools which lowers the barrier to entry of Cell Hashing workflows for small laboratories in the field of single-cell sequencing, and should be useful for core facilities that can use cell hashing to mix and overload samples to increase throughput and allow their customers to use only a fraction of one or many wells.

## Methods

### Sample processing

Biological specimens were purchased from Bloodworks Northwest as freshly drawn whole blood. All sample collections were conducted by Bloodworks Northwest under IRB-approved protocols, and all donors signed informed consent forms. PBMCs were isolated in-house using Ficoll Premium (GE Healthcare, 17-5442-03), were cryopreserved using Cryostor10 (StemCell Technologies, 07930), and stored in liquid nitrogen until use. PBMCs were thawed at 37 °C using AIM V medium (Gibco, 12055091).

### FACS

PBMCs were fluorescence activated cell sorted (FACS) into naïve T-cells (CD45+ CD3+ CD45RA+ CD27+), memory T-cells (CD45+ CD3+, excluding CD45RA+ CD27+) and a non-T-cell bulk population (CD45+ CD3−). Briefly, cells were incubated with TruStain FcX (BioLegend, 422302) for 10 min on ice, followed by staining with antibodies (Additional file 6: Table S6) for 20 min on ice. Cells were washed with AIM V medium plus 25 mM HEPES and sorted on a BD FACSAria Fusion. An aliquot of each post-sort population was used to collect 2,000 events to assess post-sort purity.

### Cell hashing

FACS sorted cells were stained according to the New York Genome Center Technology Innovation Lab protocol (v2019-02-13; https://citeseq.files.wordpress.com/2019/02/cell_hashing_protocol_190213.pdf). Briefly, one million cells of each population were resuspended in 100 μl of staining buffer: DPBS without calcium and magnesium (Corning 21-031-CM) supplemented with 2% w/v BSA (Sigma-Aldrich A2934, “PBS + BSA”). 10 μl TruStain FcX (BioLegend, 422302) was added and cells were incubated on ice for 10 min, after which they were stained with 0.5 μg of a TotalSeq-A hashing antibody (Additional file 6: Table S6) on ice for 30 min. Stained cells were washed three times with 1 mL of PBS + BSA. Cells from each population were pooled together in equal numbers and passed through a 35 μm Falcon Cell Strainer (Corning, 352235). All cell counts were performed using a Cellometer Spectrum Cell Counter (Nexcelom) using ViaStain Acridine Orange/Propidium Iodide solution (Nexcelom, C52-0106–5).

### 10x library preparation

Libraries were prepared using the Chromium Single Cell 3′ v3 reagent kit (10x Genomics, 1000075) following the 10x Genomics User Guide (CG000183 Rev A), with the only modification being cell overloading. All libraries were sequenced on an Illumina NovaSeq S4 flowcell. Target read counts were 30,000 reads per cell for RNA libraries and 2,000 reads per cell for HTO libraries.

### Data pre-processing

Raw sequencing data was converted from BCL to FASTQ format using bcl2fastq2 (Illumina v2.20.0.422, parameters: –use-bases-mask = Y28,I8,Y91, –create-fastq-for-index-reads, –minimum-trimmed-read-length = 8, –mask-short-adapter-reads = 8, –ignore-missing-positions, –ignore-missing-controls, –ignore-missing-filter, –ignore-missing-bcls, -r 18 -w 18 -p 50, –barcode-mismatches = 0). Gene expression data was processed using Cell Ranger count(10x Genomics v4.0.0) and aligned to the GRCh38 (hg38) reference genome (refdata-cellranger-atacGRCh38-1.1.0) with the option –expect-cells set to 40,000 for all wells. After running Cell Ranger count, the BarMixer Rmarkdown notebook add_tenx_rna_metadata.Rmd was used to prepare Cell Ranger outputs for downstream analysis.

### HTO counting

Hashtag counting was profiled using the Linux “time -v” command (GNU time v1.7, https://www.gnu.org/software/time/) on a Google Cloud Platform Compute Engine VM Instance with 12 vCPUs (Intel Skylake or later) and 78 GB of RAM. A list of filtered cell barcodes provided by Cell Ranger count as “barcodes.tsv” files were used as the barcode whitelist input to HTO counting software tools where necessary. BarCounter was run with default parameters. KITE (v0.0.2, https://github.com/pachterlab/kite, [4]) was run with default parameters and evaluated both single threaded and using eight threads. Cell Ranger count was run in Feature Barcode Only mode (10x Genomics v4.0.0, parameters: –nosecondary –nopreflight –disable-ui –expect-cells = 40,000). CITE-seq Count was run with default parameters including UMI correction [15], https://hoohm.github.io/CITE-seq-Count/, v1.4.3, parameters: -cbf 1 -cbl 16 -umif 17 -umil 28 -cells 40,000), as well as without UMI correction by including the additional parameter –no_umi_correction.

### HTO category assignment

Cells were assigned to individual HTO-defined samples, doublet, multiplet, or no hash categories using a multi-step process contained in the BarMixer package for R, all of which are performed in sequence for a given well using the hto_processing.Rmd script provided in BarMixer. The matrix of HTO counts per cell barcode is read from BarCounter outputs, and cutoffs for positive or negative cell barcodes are defined for each HTO separately. Cutoffs are determined by removing all counts below 10. Then, a test of unimodality is performed using the modetest function from the multimode package for R (v1.4 [1], parameters: method = “HH” and B = 20) to use the Dip Test of Unimodality [9] with 20 replicates. If the distribution is unimodal, the cutoff is set to the mean value plus 2 times the standard deviation of log-transformed values. This allows capture of some positive hashes when the distribution of hashes is not bimodal, though clear bimodal separation is ideal. If the distribution of counts is not unimodal, the values are log-transformed, and 2 center K-means clustering is performed using the base R kmeans function. Cluster centers are then compared to determine if the higher center is more than fourfold greater than the lower center. If so, the cutoff is set to the minimum value in the higher cluster. Otherwise, the cutoff is set to the maximum value of all cell barcodes, and no barcodes are considered passing. After setting a cutoff for each HTO, cell counts are converted to a binary matrix of passing (1 = greater than or equal to the cutoff) or failing (0 = less than the cutoff) values, and the number of passing values are counted for each cell barcode. Cells with a single passing value are assigned to "singlets", two passing values to "doublets", more than two passing values to "multiplets", and no passing values to "no hash" categories. This information is used to generate a table of hashing categories and the HTO barcode(s) assigned to each cell barcode.

### Splitting and merging data by sample

After performing HTO category assignment for each well, a second script in the BarMixer package, split_h5_by_hash.Rmd, is used to split singlet cells from each sample and from non-singlet categories. This script reads both the HTO category assignment table generated above for each well and the HDF5-formatted count matrix generated by Cell Ranger (10x Genomics). For each well, this script generates a separate HDF5 file for each sample per well. After performing this split step for each well in the experiment, a third script from BarMixer, merge_h5_by_hash.Rmd, assembles the HDF5 files for each sample across all wells into a single HDF5 output, and uses the combined information from these files to generate a comprehensive QC report for data from all wells. All steps for category assignment, splitting, and merging can be performed using wrapper script provided in the BarWare-pipeline repository, 02_run_BarMixer.sh, available at https://github.com/AllenInstitute/BarWare-pipeline.

### RNA-seq analysis

Merged HDF5 files from the final step of the BarWare pipeline were used as input and analyzed using Seurat (v4.0.3 [8]). Singlet data was read using the BarMixer (v1.2.0) function read_h5_seurat and merged into a single Seurat Object . Low quality barcodes and extreme outliers were filtered out by subsetting barcodes with less than 25% mitochondrial counts, RNA UMI counts of at least 1000 and less than 25,000, and at least 500 genes detected. We normalized the data using the Seurat function SCTransform [6], performed dimensionality reduction using the RunPCA function, generated a two-dimensional UMAP projection from the first 50 principal components using the RunUMAP function, and clustered the cells using the first 50 principal components using the FindNeighbors and FindClusters functions. We mapped our dataset to a reference PBMC CITE-seq dataset from [8] using the FindTransferAnchors function (parameters: dims = 1:50) and transferred cell type labels from the reference to our dataset using the MapQuery function.

### Data analysis and visualization software

Visualization of HTO profiling results and gene expression data was performed using R v.3.6.3 and greater [13] in the Rstudio IDE or using the Rstudio Server Open Source Edition [16] as well as the following packages: for data visualization, ggplot2 [18], cowplot [20], ggrastr [12], pheatmap [10]; for general data analysis and manipulation, dplyr [19], data.table [2], and janitor [3]; for scRNA-seq data analysis, Seurat [8]. Comparison of barcode classifications between HTO counting tools was performed using Python (v3.7.3) and the Pandas module [14].

## Availability and requirements

**Project name:** BarWare pipeline

**Project home page:** https://github.com/AllenInstitute/BarWare-pipeline

**Operating system(s):** UNIX/Linux operating systems.

**Programming language:** C, R, and bash

**Other requirements:** R v3.6.3 or higher

**License:** Allen Institute Software License (modified 2-clause BSD license)

**Any restrictions to use by non-academics:** redistribution and use for commercial purposes restricted without further permission.

## Supplementary Information

**Additional file 1: Table S1.** HTO category agreement across wells. Fraction of agreement of cell barcode assignment to each HTO category for each pooled sample well based on BarCounter and CITE-seq-Count processing. Well: Pooled sample well. Frac_singlet: Fraction of singlet calls that agree using BarCounter and CITE-seq-Count. Frac_doublet: Fraction of doublet calls that agree using BarCounter and CITE-seq-Count. Frac_multiplet: Fraction of multiplet calls that agree using BarCounter and CITE-seq-Count. Frac_no-hash: Fraction of no hash detected calls that agree using BarCounter and CITE-seq-Count.

**Additional file 2: Table S2.** Barcode category assignment discrepancies. Counts and count-derived metrics obtained for each of the top two hashes are shown for each cell barcode assigned to the singlet category BarCounter results. but considered a doublet based on CITE-Seq-Count results. Well: Pooled sample well. Barcode: Cell barcode. BarCounter_1st: Counts for the highest-scoring hash based on BarCounter. BarCoutner_2nd: Counts for the second highest-scoring hash based on BarCounter. CITE_1st: Counts for the highest-scoring hash based on CITE-seq-Count. CITE_2nd: Counts for the second highest-scoring hash based on CITE-seq-Count. Change_1st: Difference in counts for the highest-scoring hash (BarCounter_1st—CITE_1st). Change_2nd: Difference in counts for the second highest-scoring hash (BarCounter_2nd—CITE-seq-Count_2nd). Prop_Change_1st: Difference in counts for the highest-scoring hash as a proportion of BarCounter counts (BarCounter_1st—CITE_1st) / BarCounter_1st. Prop_Change_2nd: Difference in counts for the second highest-scoring hash as a proportion of BarCounter counts (BarCounter_2nd—CITE_2nd) / BarCounter_2nd. Ratio_1st:2nd: Ratio of the highest-scoring BarCounter counts to the second high-scoring BarCounter counts (BarCounter_1st / BarCounter_2nd).

**Additional file 3: Table S3.** Sequenced read count statistics. Library ID: Pooled library ID. # Cells: Number of cells loaded. Reads: Number of sequenced read trios for each library (I1, R1, and R2). Reads per cell: Mean number of sequenced reads per cell barcode.

**Additional file 4: Table S4.** Benchmarking statistics for HTO counting methods. Tool: Software tool used for benchmarking. Well: Pooled sample well. Elapsed Time (h:mm:ss): Elapsed (clock) time passed to analyze each well. User Time (s): User Time elapsed to analyze each well. % CPU: Maximum CPU load during well analysis. Max Resident Set Size (KB): Maximum resident memory set size during well analysis. Output Size (bytes): Output file size after analysis.

**Additional file 5: Table S5.** BarWare HTO category assignment counts. Well: Pooled sample well. Total Barcodes: Number of cell barcodes identified for each well. Singlet: Number of cell barcodes assigned to the singlet category. Doublets: Number of cell barcodes assigned to the doublet category. Multiplets: Number of cell barcodes assigned to the multiplet category. No Hash: Number of cell barcodes assigned to the no hash detected category.

**Additional file 6: Table S6.** Antibodies used for Cell Hashing and FACS. Manufacturer: Reagent manufacturer. Catalog No.: Manufacturer catalog number. Conjugate: Moiety (fluorophore or oligonucleotides) conjugated to each antibody. Target: Antibody binding target(s). Clone: Antibody clone. Vol per M cells (µL): Volume of antibody added per million cells for staining.

## Data Availability

Raw data is deposited in the NCBI Database of Genotypes and Phenotypes (dbGaP, Accession ID: phs002695.v1) for controlled access. Processed data has been deposited in the NCBI Gene Expression Omnibus database (GEO, Series Accession ID: GSE181862). Code and documentation for the BarcodeTender pipeline, including BarCounter and BarMixer, are available on Github at https://github.com/AllenInstitute/BarWare-pipeline. Code used for benchmarking and figure generation in this manuscript are available on Github at https://github.com/AllenInstitute/BarWare-manuscript. A demonstration dataset with scripts for running the BarWare pipeline and all output files are available in the Zenodo repository at https://zenodo.org/record/5620859.

## Abbreviations

CellBC: Cell Barcode
CSV: Comma Separated Values
FACS: Fluorescence Activated Cell Sorting
GEO: Gene Expression Omnibus
HDF5: Hierarchical Data Format 5
HTO: Hash Tag Oligonucleotide
JSON: JavaScript Object Notation
KITE: Kallisto Indexing and Tag Extraction
IRB: Institutional Review Board
PBMC: Peripheral Blood Mononuclear Cell
QC: Quality Control
scRNA-seq: Single-cell ribonucleic acid sequencing
UMAP: Uniform manifold approximation and projection
UMI: Unique Molecular Identifier
UUID: Universally Unique Identifier

## References

[1] Ameijeiras-Alonso J, Crujeiras RM, Rodríguez-Casal A. Multimode: an R package for mode assessment. 2018. arXiv [stat.CO]. arXiv. http://arxiv.org/abs/1803.00472.

[2] Dowle M, Srinivasan A. 2020. Data.table: extension of ‘data.frame’. https://CRAN.R-project.org/package=data.table.

[3] Firke S. 2020. Janitor: simple tools for examining and cleaning dirty data. https://CRAN.R-project.org/package=janitor.

[4] Gehring J, Park JH, Chen S, Thomson M, Pachter L (2020). Highly multiplexed single-cell RNA-Seq by DNA oligonucleotide tagging of cellular proteins. Nat Biotechnol.

[5] Genge PC, Roll CR, Heubeck AT, Swanson E, Kondza N, Lord C, Weiss M, Hernandez V, Phalen C, Thomson Z, Torgerson TR, Skene PJ, Bumol TF, Reading J (2021). Optimized workflow for human PBMC multiomic immunosurveillance studies. STAR Protoc.

[6] Hafemeister C, Satija R (2019). Normalization and variance stabilization of single-cell RNA-Seq data using regularized negative binomial regression. Genome Biol.

[7] Haining WN, Ebert BL, Aravind Subrmanian E, Wherry J, Eichbaum Q, Evans JW, Mak R (2008). Identification of an evolutionarily conserved transcriptional signature of CD8 memory differentiation that is shared by T and B cells. J Immunol.

[8] Hao Y, Hao S, Andersen-Nissen E, Mauck WM, Zheng S, Butler A, Lee MJ (2021). Integrated analysis of multimodal single-cell data. Cell.

[9] Hartigan JA, Hartigan PM (1985). The dip test of unimodality. Ann Stat.

[10] Kolde R. 2019. Pheatmap: pretty heatmaps. https://CRAN.R-project.org/package=pheatmap.

[11] Macosko EZ, Basu A, Satija R, Nemesh J, Shekhar K, Goldman M, Tirosh I (2015). Highly parallel genome-wide expression profiling of individual cells using nanoliter droplets. Cell.

[12] Petukhov V, van den Brand T, Biederstedt E. 2020. Ggrastr: Raster layers for “ggplot2.” https://CRAN.R-project.org/package=ggrastr.

[13] R Core Team (2021). R: a language and environment for statistical computing.

[14] Reback J, jbrockmendel, McKinney W, Van den Bossche J, Augspurger T, Cloud P, Hawkins S, et al. Pandas-Dev/pandas: Pandas 1.3.2. Zenodo. 2021. 10.5281/ZENODO.3509134.

[15] Roelli P, bbimber, Flynn B, santiagorevale, Gui G. Hoohm/CITE-Seq-Count: 1.4.2. 2019. 10.5281/zenodo.2590196.

[16] RStudio Team (2020). RStudio: integrated development environment for R.

[17] Stoeckius M, Zheng S, Houck-Loomis B, Hao S, Yeung BZ, Mauck WM, Smibert P, Satija R (2018). Cell hashing with barcoded antibodies enables multiplexing and doublet detection for single cell genomics. Genome Biol.

[18] Wickham H (2016). ggplot2: elegant graphics for data analysis.

[19] Wickham H, François R, Henry L, Müller K. Dplyr: a grammar of data manipulation. 2020. https://CRAN.R-project.org/package=dplyr.

[20] Wilke CO. Cowplot: streamlined plot theme and plot annotations for “ggplot2.” 2020. https://CRAN.R-project.org/package=cowplot.

[21] Zheng GXY, Terry JM, Belgrader P, Ryvkin P, Bent ZW, Wilson R, Ziraldo SB (2017). Massively parallel digital transcriptional profiling of single cells. Nat Commun.

